# Late Surgical Publications

**Published:** 1845-07-01

**Authors:** 


### LATE SURGICAL PUBLICATIONS.

New Elements of Operative Surgery, by Alf. A. L. M. Velpeau, Surgeon to La Charite, Paris. Translated by P. S. Townsend, M. D., New-York; notes by Dr. Mott, with several hundred pages of entire new matter, &c., &c., in 3 vols. 8vo., 1845. (From the publisher, Henry G. Langley, N. Y.)

With this work in the original we were already somewhat familiar, and have believed it to be one of the most complete systems of Operative Surgery extant; containing as it does, not only the authors own, but also all other modes of operation, which, either from the ingenuity or reputation of the inventors, have any just claim to notice.

In most of the views of Velpeau upon Surgical practice, the American readers will find no ground of difference. Yet upon the great point of "Union by First or Second Intention" after large amputations, a difference of opinion certainly does exist; nor shall we be prepared to adopt his peculiar treatment of all fractures by "dextrine." It will however, be seen, that upon neither of these points is his practice without argument or defence.

The American edition, of which but one volume has yet been issued, is greatly enlarged from the original by "notes and additions," not *written* (as we were led to infer they were from the title page) by Dr. Mott, but written and compiled by Dr. Townsend, and *approved* by Dr. Mott; yet they are none the less valuable. F. H. H.

---

The Principles of Surgery, by James Miller, Prof. of Surgery, in the University of Edinburgh, Surgeon to the Royal Infirmary, &c., &c., 1 vol., 8vo., 1845. (From the publishers, Lea & Blanchard, Philadelphia.)

This is the last from that inexhaustable fountain of surgical authors, Edinburgh. Lizars, Symes, Liston, Ferguson, and Miller, have followed in rapid succession, and now scarcely has this last been announced from the English press, when an American edition is before us.

We have examined the work carefully, scarcely expecting however upon the "elements of surgery" any thing new. In this we have been greatly disappointed It supplies much that was needed in surgical pathology, a department of surgery hitherto almost entirely overlooked by our surgical writers. The author is also evidently a sound, practical man, and to all such readers it will not fail to recommend itself. F. H. H.

---

The first lines of the Theory and Practice of Surgery, including the principal operations, by Samuel Cooper, of University College Hospital, London; with notes and additions by Willard Parker, of the College of Physicians and Surgeons, New-York, 2 vols., 8vo. (From the publishers, S. & W. Wood, N. Y.)

We were delighted to see this new and beautiful edition of our old friend "Cooper's First Lines." The last (seventh English edition) had received from Mr. Cooper most of those additions which the modern improvements of Surgery had rendered necessary, and under the finishing hand of Dr. Parker, it is now made complete. The names of S. Cooper and Parker are alone a sufficient guarantee of the value of the volumes.
F. H. H.

